# A New Method for Bearing Fault Diagnosis across Machines Based on Envelope Spectrum and Conditional Metric Learning

**DOI:** 10.3390/s24092674

**Published:** 2024-04-23

**Authors:** Xu Yang, Junfeng Yang, Yupeng Jin, Zhongchao Liu

**Affiliations:** 1 School of Intelligent Manufacturing, Nanyang Institute of Technology, Nanyang 473004, China; yangxuican@bjtu.edu.cn (X.Y.); lzc@nyist.edu.cn (Z.L.); 2 School of Electrical Engineering, Beijing Jiaotong University, Beijing 100044, China; 3 School of Aerospace, Mechanical and Mechatronic Engineering, The University of Sydney, Sydney, NSW 2050, Australia

**Keywords:** fault diagnosis, conditional metric learning, envelope spectrum, convolutional neural network

## Abstract

In recent years, most research on bearing fault diagnosis has assumed that the source domain and target domain data come from the same machine. The differences in equipment lead to a decrease in diagnostic accuracy. To address this issue, unsupervised domain adaptation techniques have been introduced. However, most cross-device fault diagnosis models overlook the discriminative information under the marginal distribution, which restricts the performance of the models. In this paper, we propose a bearing fault diagnosis method based on envelope spectrum and conditional metric learning. First, envelope spectral analysis is used to extract frequency domain features. Then, to fully utilize the discriminative information from the label distribution, we construct a deep Siamese convolutional neural network based on conditional metric learning to eliminate the data distribution differences and extract common features from the source and target domain data. Finally, dynamic weighting factors are employed to improve the convergence performance of the model and optimize the training process. Experimental analysis is conducted on 12 cross-device tasks and compared with other relevant methods. The results show that the proposed method achieves the best performance on all three evaluation metrics.

## 1. Introduction

Bearings are among the most often used elements in mechanical devices, serving the function of supporting and carrying rotating shafts [1,2,3]. Bearings play a crucial role in mechanical equipment as they reduce friction and wear, ensuring stable operation of the equipment. Therefore, bearing fault diagnosis is of vital importance in maintaining the normal functioning of mechanical equipment [4,5,6].

Currently, there are numerous approaches available for bearing fault diagnosis. Vibration analysis technology is one of the most widely used, with its core function being to detect bearing vibration signals and judge whether there are faults in the bearings based on signal characteristics. This technology has become the preferred choice due to its efficiency. Traditionally, fault diagnosis methods focus on the analysis of pulse impact intervals in vibration signals to distinguish different fault types. In the contemporary landscape of fault diagnosis, an array of algorithms have been developed based on the principles of mechanical fault theory. These methods encompass diverse techniques, such as resonance demodulation [7], envelope demodulation [8,9], generalized demodulation [10], and order ratio analysis [11]. The recent surge in the field of bearing fault diagnosis can be attributed to the continuous advancements in deep-learning technologies. This evolution has led to the validation and widespread adoption of innovative methods, including convolutional neural networks [12], autoencoders [13], recurrent neural networks [14], generative adversarial networks [15], and graph neural networks [16]. It is noteworthy that these models have stringent requirements for data, which require the data distribution of the training set and the test set to remain consistent. However, in practical engineering applications, due to changes in rotation speed, load, and sensor installation position, the data of the training set and test set may experience shifts. Therefore, unsupervised fault diagnosis approaches based on transfer learning have emerged. These methods can be divided into two major categories according to different application scenarios: unsupervised cross-domain learning on the same device and cross-domain learning on different devices. The emergence of this method provides a new approach to overcoming data drift issues, making fault diagnosis more feasible.

The scenario addressed by unsupervised domain adaptation within the same device is when the source domain and target domain data come from different vibration data under varying rotational speeds or loads. Many scholars have proposed numerous solutions to tackle the cross-domain fault diagnosis problem. Li et al. [17] constructed a deep convolutional neural network and used the maximum mean discrepancy based on multiple kernels (MK-MMD) to reduce the domain feature distance between multiple layers of the neural network, significantly improving the diagnostic performance. The method was validated using training bogie-bearing data. Chen et al. [18] employed domain adversarial training techniques to minimize the differences between the source domain and target domain data. They applied this approach in a deep transfer convolutional neural network and conducted extensive domain shift experiments on gearbox and bearing datasets. Li et al. [19] addressed the issue of low diagnostic accuracy due to insufficient training data by utilizing deep generative models to synthesize fault signals under the condition of known healthy data. The generated fault signals were then used in the domain adaptation process and validated for effectiveness using two different bearing fault datasets. Xiao et al. [20] utilized simulated fault mechanism data to construct a data- and physics-coupled fault diagnosis model, reducing the dependence on experimental setups. The proposed improved Joint Maximum Mean Discrepancy (JMMD) simultaneously aligned the conditional distribution and marginal distribution. The results showed that the proposed method achieved unsupervised domain adaptive fault diagnosis. In the scenario where the fault categories differ between the source and target domain datasets, Han et al. [21] proposed an intrinsic and extrinsic domain generalization network. This network combined label loss, triple loss, and adversarial loss functions to achieve gearbox fault diagnosis in unseen operating conditions within the target domain.

The scenario addressed by unsupervised domain adaptation across different devices is when the source domain and target domain data come from different devices’ vibration data. Some scholars have proposed feasible solutions. Guo et al. [22], employing transfer learning techniques and adversarial training, introduced a deep convolutional transfer learning network (DCTLN) that adeptly diagnosed bearing faults across three disparate devices. Liu et al. [23] considered both rotational speed shifts and cross-device fault diagnosis tasks and proposed a deep adversarial subdomain adaptive network (DASAN). Experimental results demonstrated the effectiveness of DASAN. Wang et al. [24] proposed a subdomain adaptive transfer learning network (SATLN) by taking into account adaptive marginals and conditional distributional bias and incorporating dynamic weighting elements. This network was validated to achieve an average diagnostic accuracy of 90.19%. It is worth mentioning that in addition to cross device scenarios, Yu et al. provided excellent fault diagnosis methods from three aspects: incremental learning [25], model interpretability [26], and an online fault diagnosis system based on an integrated learning strategy [27].

The above research results indicate that current cross-domain fault diagnosis within the same device can achieve high diagnostic effectiveness. However, methods for cross-device fault diagnosis face significant deviations between the source domain and target domain data. Moreover, most models ignore discriminative information under marginal distribution, resulting in subpar diagnostic accuracy. In response to the multifaceted challenges at hand, this research introduces a pioneering methodology designed to diagnose bearing faults across a spectrum of devices. The essence of this methodology lies in the amalgamation of envelope spectrum analysis and the sophisticated principles of conditional metric learning. Primarily, the methodology undertakes the transformation of temporal vibration signals into their frequency-domain manifestations through the adept application of envelope spectrum analysis. Subsequently, a cutting-edge convolutional neural network model is meticulously crafted, incorporating a deep Siamese transfer approach, while being intricately grounded in the foundational principles of conditional metric learning. This innovative framework not only enhances the diagnostic accuracy of bearing faults but also showcases a nuanced understanding of the intricate interplay between envelope spectrum analysis and conditional metric learning. Finally, the proposed method is validated in six domain adaptation tasks across three different devices and compared with current advanced cross-device fault diagnosis methods. The main innovations are as follows:

(1) In order to address the issues of data discrepancies and domain biases in cross-device fault diagnosis, we innovatively introduced envelope spectrum analysis in our research. This method aims to reduce the differences in data generated by different devices at the frequency domain level, thereby optimizing data consistency and enhancing the expression of fault characteristics.
(2) We adopted a feature transfer strategy based on the conditional kernel Bures metric, which further reduces the biases between data from different domains and provides a solid foundation for precise training of diagnostic models.
(3) To enhance the optimization of the training process and the accuracy of diagnosis, we implemented dynamic weight learning technology. This technology adjusts the weight distribution in real-time during the learning process to respond to the importance of different categories and features, ensuring that the model achieves optimal performance in various fault diagnosis tasks.
(4) To comprehensively demonstrate the effectiveness of our proposed method, we conducted cross-device fault diagnosis research on two public bearing fault datasets and one private dataset. We carefully designed six different diagnostic tasks and compared five advanced fault diagnosis methods using three quantitative metrics. Through this rigorous experimental design and evaluation, our method demonstrated its effectiveness and superiority in various tasks.

The chapters are arranged as follows: Section 2 provides the definition of the fault diagnosis problem discussed in this article. Section 3 discusses the fault diagnosis methods employed in this study. Section 4 presents the results of fault diagnosis and compares the performance of the proposed method. Finally, Section 5 provides a comprehensive summary of the entire study.

## 2. Problem Formulation

The pivotal objective is to discern and classify the fault states of varied devices, distinguishing between normal operational states and those indicative of faults. Notwithstanding, practical implementation encounters formidable hurdles, notably the paucity of labeled samples specific to the target domain. To surmount this challenge, a strategic recourse involves harnessing the available labels from the source domain. This proactive approach serves as the bedrock for constructing a robust fault diagnosis model, proficient in extrapolating and predicting the labels associated with the target domain’s data. This adaptive methodology enhances the applicability and efficacy of bearing fault diagnosis in real-world scenarios.

Data $D_{S}^{h\_s}={\left\{(x_{s}^{\left(i\right)},y_{s}^{\left(i\right)})\right\}}_{i=1}^{N}$ from device A constitute the source domain, while data $D_{T}^{h\_t}={\left\{(x_{t}^{\left(i\right)})\right\}}_{i=1}^{M}$ from device B constitute the target domain. *N* and *M* quantitatively represent the samples within the source and target domains. Health states are distinguished as *h_s* and *h_t*. Labels ${\left\{(y_{s}^{\left(i\right)})\right\}}_{i=1}^{N}$ are accessible for the $D_{S}^{h\_s}={\left\{(x_{s}^{\left(i\right)})\right\}}_{i=1}^{N}$, while the data $D_{T}^{h\_t}={\left\{(x_{t}^{\left(i\right)})\right\}}_{i=1}^{M}$ lack such annotations. There are significant differences in the distribution of P and Q data, leading to the occurrence of a domain shift P≠Q. This paper undertakes the challenge of formulating an unsupervised fault diagnosis model. The focus is on addressing cross-device fault diagnosis tasks, specifically from device A to device B (target domain).

## 3. Methods

In this dedicated section, we meticulously expound upon the foundational tenets governing envelope spectrum analysis, a pivotal facet in the realm of fault diagnosis. The ensuing discourse delves into the intricacies of our meticulously crafted deep Siamese convolutional neural network model. Subsequently, we introduce the theoretical underpinnings of conditional kernel Bures (CKB), a sophisticated framework augmenting our analytical prowess. Following this, a comprehensive exploration of the dynamic weighting mechanism ensues, contributing to the nuanced understanding of our proposed methodology.

### 3.1. Envelope Spectrum

Envelope spectrum analysis is a commonly used signal analysis method in mechanical fault diagnosis. By performing envelope analysis on vibration signals, periodic components in mechanical systems can be effectively extracted. 

Generally, the fault characteristic frequency in the envelope spectrum can preliminarily identify the fault type, and the formula is as follows [28]:

(1) $$\left\{\begin{array}{l} f_{0}=\frac{1}{2}Z(1-\frac{d}{D}\cos\alpha )f_{r}\\ f_{i}=\frac{1}{2}Z(1+\frac{d}{D}\cos\alpha )f_{r}\\ f_{b}=\frac{D}{2d}[1-{\left(\frac{d}{D}\right)}^{2}\cos^{2}\alpha ]f_{r}\\ f_{c}=\frac{1}{2}[1-\frac{d}{D}\cos\alpha ]f_{r} \end{array}\right.$$

where *D* is the bearing pitch diameter; *d* is the rolling element diameter; *f* is the rotation frequency; $f_{0},f_{i},f_{b},f_{c}$ represent the outer ring, inner ring, rolling element, and cages fault characteristic frequencies, respectively; and α is the contact angle between the rolling element and the raceway. 

Although traditional envelope spectrum analysis provides a feasible method for fault feature extraction, it is usually limited to linear, stationary signals. However, in practical applications, many bearing fault signals are non-stationary and contain noise, requiring more advanced analysis techniques. The method we propose is based on conditional metric learning, which exhibits better performance in analyzing non-stationary signals containing complex noise and interference because it considers the potential non-linear features related to faults in the signal. At the same time, our method also utilizes unsupervised domain adaptation techniques to optimize the model’s generalization ability in new domains (such as data from different equipment or operating conditions), which may not be achievable with envelope spectrum analysis.

This method is based on the principle of Fourier transform, decomposing the signal into multiple frequency components. Then, the amplitude variations of these frequency components are analyzed using envelope detection techniques to obtain an envelope spectrum. The original time-domain signal is transformed into a one-dimensional frequency spectrum. The analytical signal z(t) of the signal x(t) is built below:

For a single classification signal, its phase function can be written as follows:

(2) z(t)=x(t)+jH(x(t))

(3) $$a(t)=\left|z(t)\right|=\sqrt{x^{2}(t)+H^{2}(t)}$$

Subsequently, the fast Fourier transform is used to convert a(t) into a frequency domain signal, resulting in an envelope spectrum signal:

(4) $$x_{k}=\sum_{n=0}^{N-1}x_{n}e^{-i2\pi kn/N}$$

$e^{i2\pi /N}$ is a primitive N−th root of 1.

### 3.2. Deep Siamese Convolutional Neural Network

Depicted in Table 1 is the underlying framework of the deep Siamese convolutional neural network model explored in this paper. A feature extractor $G_{f}$ and classifier $G_{c}$ constitute the proposed model. The implementation of batch normalization ensures stable training dynamics by normalizing the input of each layer. Meanwhile, the activation function, ReLU, introduces non-linearity to the model, enabling it to capture complex patterns in the data. In the classifier, the presence of two fully connected layers facilitates the hierarchical learning of abstract features, contributing to the model’s discriminative capabilities. This carefully designed architecture aims to optimize the extraction of distinctive features and enhance the discriminatory power of the model.

### 3.3. Conditional Kernel Bures

The CKB, a new measure of gauging conditional distribution disparities [29,30], finds its niche within the realm of Optimal Transport (OT). Operating as a statistically grounded and interpretable tool, CKB facilitates an in-depth exploration of the intricate knowledge transfer mechanisms inherent in transfer learning models. Robust validation across the domains of computer vision and pattern recognition attests to the efficacy and interpretability of CKB. The strategic infusion of CKB into cross-device mechanical fault diagnosis stems from its distinctive interpretability and adeptness in domain adaptation. This strategic integration aims to elevate the clarity of interpretative aspects related to features within the transfer model while concurrently mitigating data disparities across a spectrum of devices.

(5) $$d_{\mathrm{CKB}}^{2}\left(R_{XX|Y}^{s},R_{XX|Y}^{t}\right)=\mathrm{tr}\left(R_{XX|Y}^{s}+R_{XX|Y}^{t}-2R_{XX|Y}^{st}\right)$$

where

(6) $$R_{XX|Y}^{st}=\sqrt{\sqrt{R_{XX|Y}^{s}}R_{XX|Y}^{t}\sqrt{R_{XX|Y}^{s}}}$$

$R_{XX|Y}$ is the conditional covariance operator, and its calculation formula is

(7) $$R_{XX|Y}=R_{XX}-R_{XY}R_{YY}^{-1}R_{YX}$$

$R_{XY}$ is the cross-covariance operator [31]

(8) $$R_{XY}=E_{XY}\left[\left(\phi (X)-\mu_{X}\right)⊗\left(\psi (Y)-\mu_{Y}\right)\right]$$

$\mu_{X}$ and $\mu_{Y}$ are the means. The non-linear mappings ϕ(X) and ψ(Y) for X and Y. Under the condition X=Y, the relationship holds $R_{XX}=R_{YY}=R_{XY}$. We opt for an equivalent transformation [30].

(9) $$\begin{array}{l} {\hat{d}}_{\mathrm{CKB}}^{2}\left({\hat{R}}_{XX|Y}^{s},{\hat{R}}_{XX|Y}^{t}\right)\\ =\mathrm{tr}\left[G_{X}^{s}{\left(\varepsilon nI_{n}+G_{Y}^{s}\right)}^{-1}\right]+\varepsilon \mathrm{tr}\left[G_{X}^{t}{\left(\varepsilon mI_{m}+G_{Y}^{t}\right)}^{-1}\right]\\ -\frac{2}{\sqrt{nm}}{\left\|{\left(H_{m}C_{t}\right)}^{T}K_{XX}^{ts}\left(H_{n}C_{s}\right)\right\|}_{*} \end{array}$$

In the domain of fault diagnosis, the variable *n* represents the sample size within the source domain, while *m* designates the corresponding quantity within the target domain. The regularization coefficient $H_{n}=I_{n}-\frac{1}{n}1_{n}1_{n}^{T}$ is denoted by ε>0. $I_{n}$ and $H_{m}$ both assume an n-dimensional form, with the latter manifesting as a diagonal matrix replete with one $1_{n}$ and the former representing an explicit kernel matrix ${\left(K_{XX}^{ts}\right)}_{ij}=k_{X}\left(x_{i}^{t},x_{j}^{s}\right)$. The kernel norm is aptly denoted as ${\left\|\cdot \right\|}_{*}$, and the interrelation between $C_{s}$ and $G_{Y}^{s}$ is succinctly expressed through the ensuing equation. This formulation establishes a foundational understanding within the realm of fault diagnosis, elucidating the crucial parameters governing the relationship between source and target domains.

(10) $$\begin{array}{l} \varepsilon n{\left(G_{Y}^{S}+\varepsilon nI_{n}\right)}^{-1}=\\ U_{s}D_{s}U_{s}^{T}=U_{s}\sqrt{D_{s}}{\left(U_{s}\sqrt{D_{s}}\right)}^{T}=C_{s}C_{s}^{T} \end{array}$$

**Figure 1:**
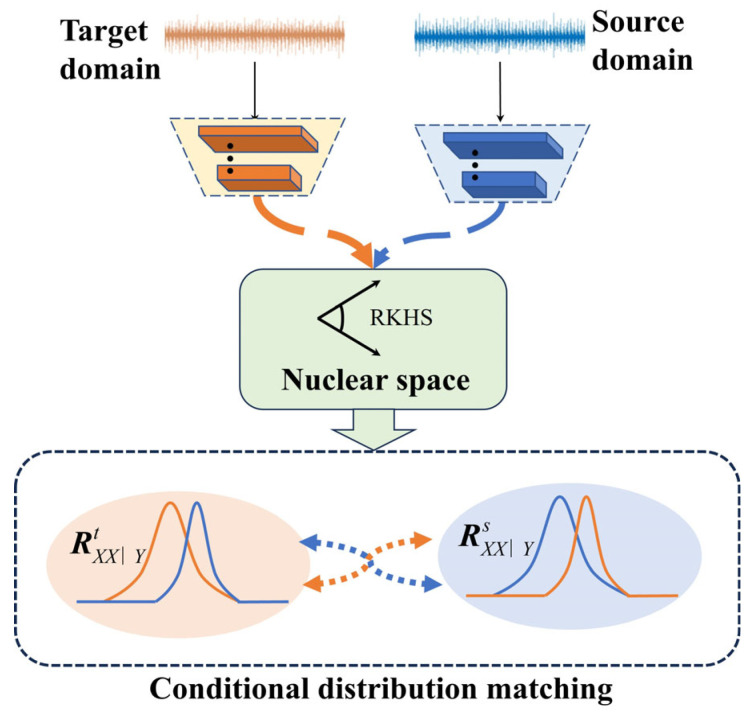
The schematic diagram of CKB.

$U_{s},D_{s}$ are the eigenvector and eigenvalue matrices. Employing the Formula (9) facilitates the quantification of the conditional distribution distance pertaining to the features of data originating from both the source and target domains. This calculated distance serves as a crucial metric for assessing the alignment between domains. Figure 1 visually presents the demonstration of the efficacy of the conditional kernel Bayes (CKB) methodology in navigating the intricacies of this conditional distribution alignment. This visualization not only reinforces the empirical findings but also provides a tangible representation of the method’s utility in the diagnostic context.

### 3.4. Dynamic Weight Mechanism

Within the domain of profound learning, the loss metric is utilized to gauge the incongruity between the prognostications of the model and the factual outcomes. Generally, the loss function’s coefficients remain unaltered, signifying uniform loss consideration for all instances. Nevertheless, instances arise where uniformity must be forsaken, compelling the incorporation of a mechanism that imbues dynamism into the weights.

The dynamic weight mechanism refers to the personalized allocation of loss weights for different samples. There are several common dynamic weight mechanisms:

(1) Category-based dynamic weight mechanism: Different weights are set for samples of different categories to adjust their contributions to the loss function. For example, larger weights can be assigned to samples of minority categories to make the model pay more attention to these samples.
(2) Difficulty-based dynamic weight mechanism: Higher weights can be assigned to samples that are more difficult, forcing the model to focus more on these challenging samples. Typically, the difficulty of a sample can be measured by the difference between its loss value and the average loss value of the training set.
(3) Adaptive learning rate-based dynamic weight mechanism: Usually, as the model continues to train, the learning rate gradually increases. Therefore, an adaptive learning rate mechanism is needed to adjust the weights of each sample.

There are two commonly used dynamic weight updating strategies, and their computation formulas are as follows:

(11) $$\lambda =\frac{2}{\exp\left(\frac{-10\;\mathrm{epoch}\;}{\max\_\mathrm{epoch}\;}\right)}-1$$

(12) $$\lambda^{*}=\frac{-4}{\sqrt{\frac{\;\mathrm{epoch}\;}{\;\max\_\mathrm{epoch}\;+1}}+1}+4$$

where, epoch is the iteration period, max_epoch is the maximum iteration period. The experimental results below indicate that the weight strategy of Formula (11) is more effective.

### 3.5. Proposed Cross-Machine Fault Diagnosis Method

#### 3.5.1. Overall Loss Function

The overall loss function during model training is a combination of three components, labeling loss, entropy loss, and domain loss. In the initial stage, we conduct supervised training utilizing data labeled within the source domain. This procedure is effectuated through the application of the label loss function.

(13) $$\begin{array}{l} L_{C}=E_{(x_{s}^{\left(i\right)},y_{s}^{\left(i\right)})\in D_{S}^{h\_s}}[-\log({\hat{y}}_{c}^{\left(n\right)})]\\ =-\frac{1}{N}\sum_{n=1}^{N}\sum_{c=1}^{C}y_{c}^{\left(n\right)}\log\frac{\exp\left({\hat{y}}_{c}^{\left(n\right)}\right)}{\sum_{\tilde{c}=1}^{C}\exp\left({\hat{y}}_{\tilde{c}}^{\left(n\right)}\right)} \end{array}$$

*N* stands for the total count of samples, while *C* denotes the number of categories. $y_{c}^{\left(n\right)}$ serves as the sign function, producing either 0 or 1. Moreover, ${\hat{y}}_{c}^{\left(n\right)}$ signifies the output value at the *c*-th node of the fully connected layer 2 for the *n*-th sample.

Next, the entropy loss function is used to constrain the output uncertainty of the target domain data. This process is unsupervised. The computation formula is as follows:

(14) $$\begin{array}{l} L_{E}=E_{(x_{t}^{\left(i\right)},y_{t}^{\left(i\right)})\in D_{T}^{h\_t}}[-\log({\hat{y}}_{c}^{\left(m\right)})]\\ =-\frac{1}{M}\sum_{m=1}^{M}\sum_{c=1}^{C}{\hat{y}}_{c}^{\left(m\right)}\log\frac{\exp\left({\hat{y}}_{c}^{\left(m\right)}\right)}{\sum_{\tilde{c}=1}^{C}\exp\left({\hat{y}}_{\tilde{c}}^{\left(m\right)}\right)} \end{array}$$

Within this framework, *M* is the overall sample count, with *C* indicating the number of categories. $y_{c}^{\left(m\right)}$ acts as the sign function, resulting in a binary outcome of 0 or 1.

Ultimately, $L_{\mathrm{CKB}}={\hat{d}}_{\mathrm{CKB}}^{2}\left({\hat{R}}_{XX|Y}^{s},{\hat{R}}_{XX|Y}^{t}\right)=0$. The approximation of these marginal distributions can be accomplished through the application of the maximum mean discrepancy loss, as formulated below.

(15) $$\begin{array}{l} L_{\mathrm{MMD}}={\hat{d}}_{\mathrm{MMD}}^{2}\left(X,Y\right)\\ =\underset{f\in F}{\sup}\left(\frac{1}{N}\sum_{i=1}^{N}f\left(x_{s}^{\left(i\right)}\right)-\frac{1}{M}\sum_{i=1}^{M}f\left(x_{t}^{\left(i\right)}\right)\right) \end{array}$$

where F is a class function in RKHS, and sup (*) represents the supremum. The overall loss function of the model is as follows:

(16) $$L_{\mathrm{all}}=L_{C}+\lambda^{*}(L_{E}+L_{\mathrm{MMD}}+L_{\mathrm{CKB}})$$

The holistic refinement of the model’s parameters is systematically achieved through the minimization of $L_{\mathrm{all}}$. This intricate optimization procedure is facilitated by the judicious application of the backpropagation algorithm in conjunction with the Ranger optimizer. Importantly, the learning rate is meticulously established at 0.002, and the comprehensive training regimen spans a predetermined 200 iterations. 

#### 3.5.2. Training and Testing Procedure of the Proposed Fault Diagnosis Framework

**Figure 2:**
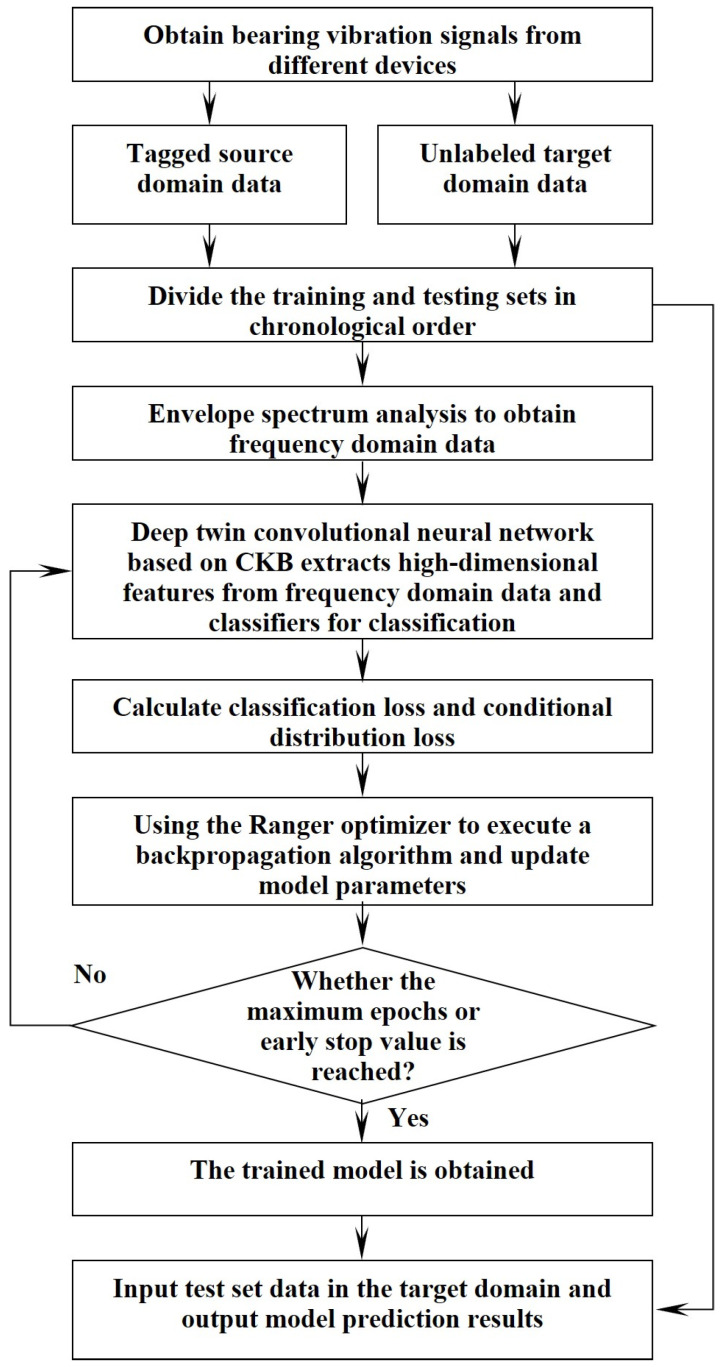
The flow chart of training and testing for the proposed framework.

Step 1: The proposed process diagram for interpretable mechanical fault diagnosis is shown in Figure 2, and the specific steps are summarized as follows.

Step 2: Obtain vibration signals from different mechanical equipment and divide the source domain and target domain data into training and testing sets in chronological order.

Step 3: Apply Hilbert envelope spectrum analysis to derive frequency domain details from both the sets designated for training and testing.

Step 4: Assemble the deep Siamese convolutional neural network, initializing the model parameters accordingly.

Step 5: Compute the overall loss function as shown in Formula (16), utilize the Ranger optimizer to perform the backpropagation algorithm, and update the model parameters.

Step 6: Execute the training process iteratively, culminating in the production of the trained model as the final outcome.

Step 7: Input the testing data from the target domain into the trained model and obtain the model’s predicted results for the health condition of the bearings.

## 4. Results

**Figure 3:**
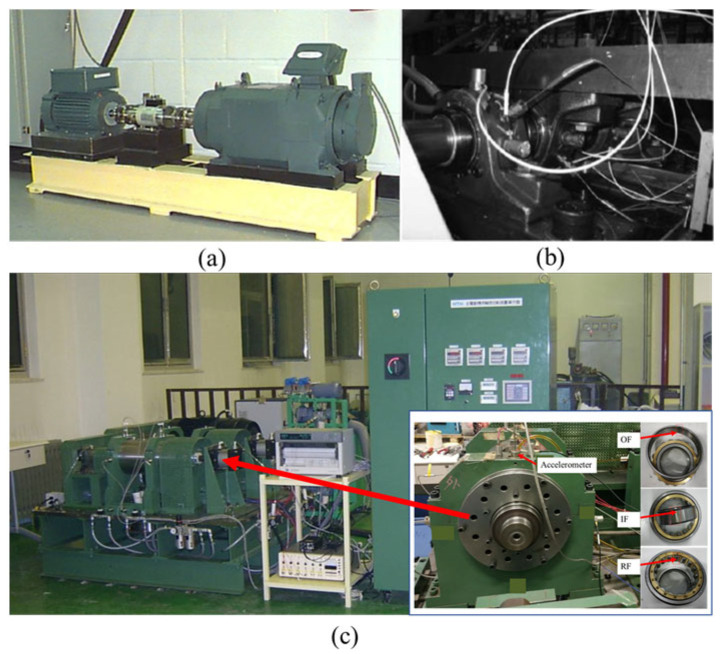
Data collection platform for (**a**) Case Western Reserve University, (**b**) NASA center’s comprehensive bearing dataset, and (**c**) high-speed traction motor bearing failure data.

Our primary aim is to substantiate the validity of the proposed methodology for cross-device fault diagnosis. The initial step involves a meticulous introduction to three distinct datasets integral to the cross-device fault diagnosis task, each containing vibration signals from motor bearings, as shown in Figure 3. Following the dataset introduction, a detailed exposition of the outcomes derived from both the proposed methodology and comparative approaches is presented, emphasizing the evaluation across three key metrics. To enhance comprehension, visualizations are employed to articulate diagnostic results derived from different methodologies. To delve deeper into the method’s intricacies, ablation experiments are conducted, providing a nuanced understanding of its operational effectiveness. This approach not only contributes to the validation of the proposed methodology but also aligns with the conventions of the scholarly literature.

### 4.1. Dataset Introduction

(1) Dataset A: This set, procured from Case Western Reserve University, comprises vibration signals obtained from an acceleration sensor with a 12 kHz sampling frequency. The signals correspond to a motor with drive-end bearings and are categorized under normal, outer race fault, inner race fault, and rolling element fault conditions. Operating at 1750 r/min and sustaining a 2 HP load, the motor provides a rich dataset for analysis.
(2) Dataset B: Sourced from the NASA center’s comprehensive bearing dataset, this dataset includes original vibration signals captured using a 20 kHz sampling frequency sensor. It likewise contains four health states. The motor’s operational parameters are set at 2000 r/min rotation speed and a 26.6 kN load, with the unique characteristic that all data points were collected under conditions of severe failure.
(3) Dataset C: the high-speed traction motor bearing failure data, again, have four health states and the data details are shown in Table 2.

### 4.2. Comparative Methods and Experimental Settings

In the validation of our proposed methodology, a comparison is undertaken with five pre-existing techniques.

(1) Initially, the deep convolutional transfer learning network (DCTLN) distinguishes itself as a pioneering strategy for cross-device domain-adaptive fault diagnosis. It amalgamates domain adversarial training with the notion of maximum mean discrepancy (MMD).
(2) The Deep Adversarial Subdomain Adaptation Network (DASAN) achieves enhanced diagnostic accuracy through the utilization of a specialized loss function designed for subdomain adaptation.
(3) Based on the fault diagnosis model proposed in this paper, we modify the domain adaptation loss function to MMD, JMMD, and LMMD, forming comparative methods (3)–(5).

In the realm of experimental design, our cross-validation experiments span three meticulously chosen datasets, yielding six distinct cross-device transfer tasks: A→C, B→A, B→C, C→A, and C→B. On average across the five experiments, each health condition encompasses 1000 samples, each with a sequence length of 1200 points. The chronological division of training and testing sets adheres to a split ratio of 0.5. Of paramount importance is the meticulous preservation of equity in our comparative study. To this end, we faithfully adhere to the original parameter settings of DCTLN and DASAN as delineated in their respective papers. Concurrently, methods (3)–(5) align with the parameter configurations meticulously detailed in our paper. Specifically, the parameters and hyperparameters of our proposed method are meticulously specified: employing the Ranger optimizer to iteratively compute optimal model parameter values corresponding to the minimum loss function, with a learning rate of 2 × 10^−3^, an L2 weight decay coefficient of 5 × 10^−3^, a batch size of 128, and a total of 200 iterations.

### 4.3. Evaluation Metrics

In this paper, three evaluation metrics are used to quantify the diagnostic performance of different methods: the mean accuracy for fault identification (Acc), the F1-score (F1), and the average area under the receiver operating characteristic curve (AUC).

(17) $$\mathrm{Acc}=\frac{TP+TN}{TP+TN+FP+FN}$$

(18) $$F1=\frac{2TP}{2TP+FP+FN}$$

*TP* (true positive), *TN* (true negative), *FP* (false positive), and *FN* (false negative) signify the relevant parameters.

### 4.4. Cross-Machine Diagnostic Results

**Figure 4:**
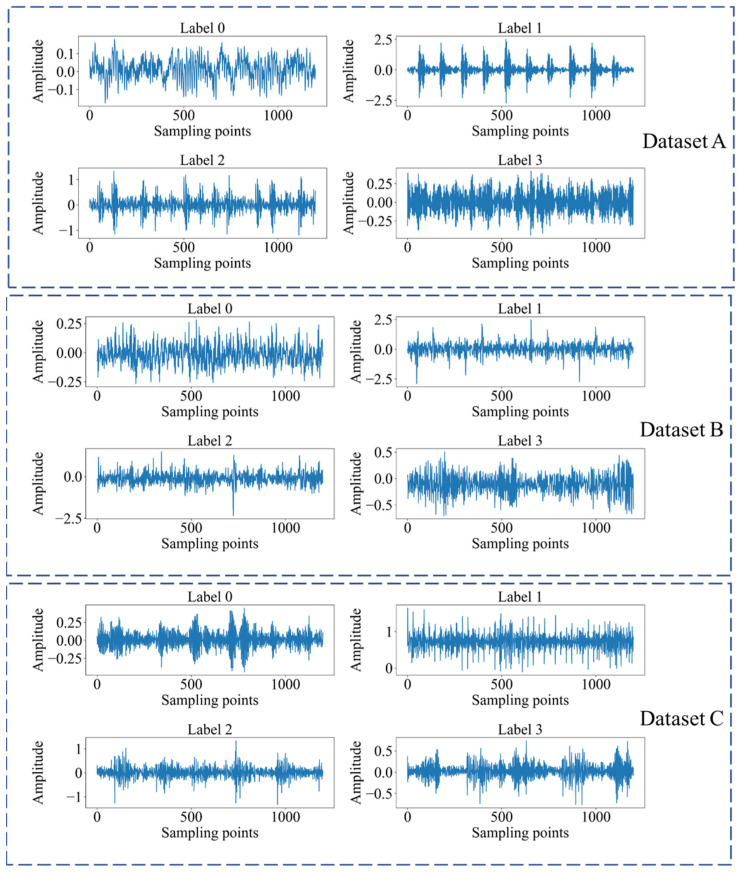
The time domain waveforms of bearing datasets.

Figure 4 displays the time-domain plots of vibration signals under different health conditions in three datasets. Through careful observation, it can be noticed that for each condition, the raw time-domain data exhibit unique vibration waveform patterns. These patterns are important indicators for understanding and monitoring the health status of the equipment, as they are directly related to the operational status of mechanical components and can provide early indications of possible equipment performance degradation. However, solely relying on visual inspection of these plots is insufficient to clearly define the corresponding vibration signals among different devices and their respective fault states. The observed differences in signal waveforms provide us with a preliminary basis for fault detection, but to improve diagnostic accuracy, further analysis and more sophisticated algorithms are needed to interpret these differences. 

**Figure 5:**
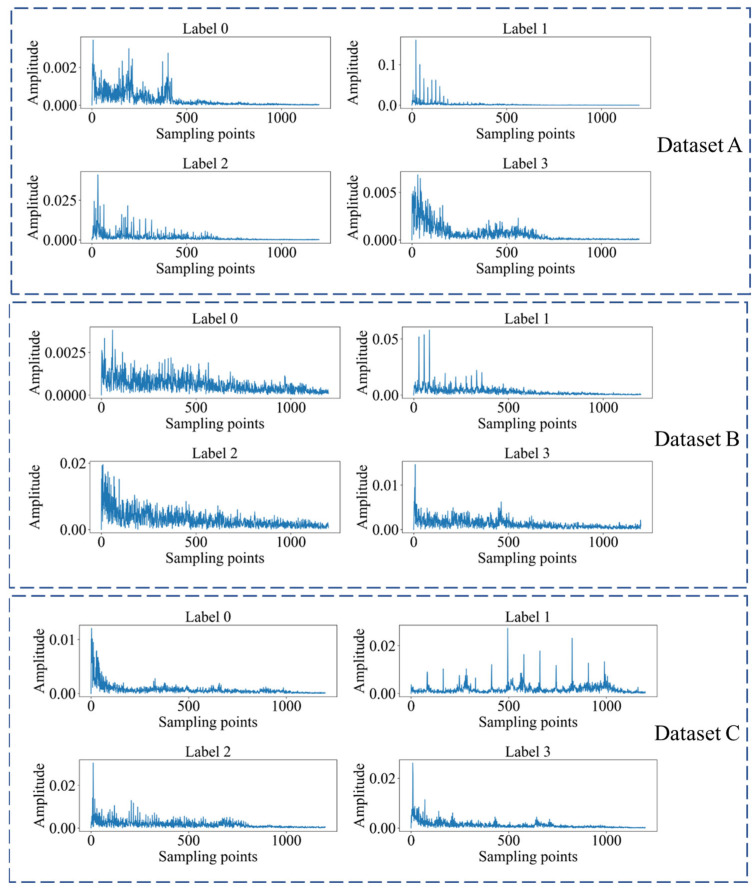
The frequency domain waveforms of bearing datasets.

In Figure 5, we present frequency-domain plots obtained through envelope spectrum analysis. Compared to time-domain data, frequency-domain plots provide a different perspective for observing the characteristics of vibration signals. Frequency domain analysis transforms time-series signals into displays of frequency components, allowing for clearer visualization of subtle cyclic variations, thereby revealing deeper mechanical fault indications. Figure 5 reveals the characteristic frequencies and energy distributions of vibration signals under different conditions in the frequency domain, which are crucial for identifying the types of faults. However, similar to the situation in Figure 4, although Figure 5 provides clearer signal differentiation, there is still insufficient direct correspondence between data with the same label from different devices. Therefore, using either time-domain or frequency-domain analysis alone is not sufficient; it is necessary to combine advanced domain adaptation techniques such as conditional metric learning to achieve high-accuracy cross-device fault diagnosis.

Comparative studies were conducted using the proposed method and five other comparison methods. Each method was tested five times for each cross-device fault diagnosis task, and the mean and variance of the three-evaluation metrics were calculated. The tabulated data in Table 3 delineate the empirical outcomes derived from our rigorous experiments. The discerning feature of paramount significance is the exemplary diagnostic proficiency demonstrated by the proposed methodology across the comprehensive spectrum of the six designated tasks. It merits explicit acknowledgment that the proposed approach attains a pristine diagnostic accuracy of 100% in tasks entailing the transition from B to A and C to A. This accomplishment underscores the robustness and efficacy of the introduced diagnostic framework, thus substantiating its applicability in diverse scenarios within the field.

**Figure 6:**
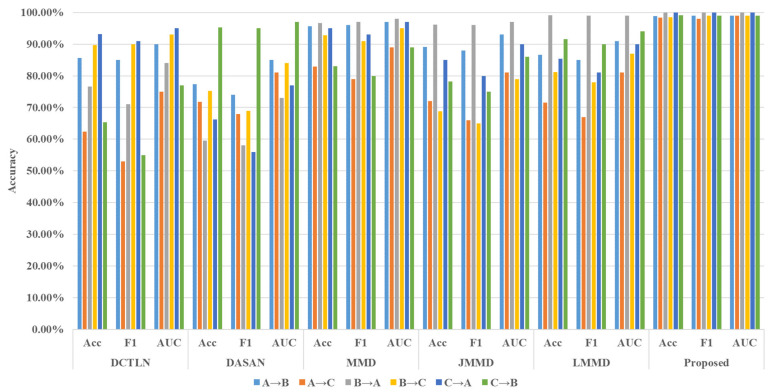
The histogram of diagnostic results.

Although DASAN considers fine-grained and discriminative features, it does not take into account discriminative information under marginal distribution. However, DASAN’s overall performance is close to that of DCTLN. This may be due to the fact that different methods perform differently on different datasets, which reflects the matching between data and models. Similarly, the stability and generalizability of methodologies (3)–(5) based on MMD, JMMD, and LMMD exhibit notable variabilities. These empirical findings underscore the pronounced efficacy of the advanced cross-device fault diagnosis methodology proposed in this study. A comprehensive overview of the methodologies’ performances across the three designated evaluation metrics is shown in Figure 6. The graphical representation unmistakably elucidates the markedly superior diagnostic outcomes attained by the innovative approach delineated in this research. The proposed methodology not only excels in diagnostic precision but also showcases a heightened robustness, offering a substantial contribution to the field of fault diagnosis in diverse device environments.

### 4.5. Visualization Analysis

**Figure 7:**
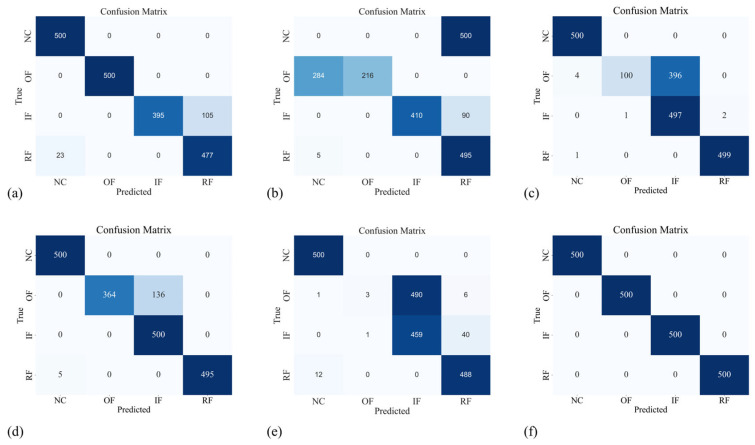
The confusion matrix results of: (**a**) DCTLN; (**b**) DASAN; (**c**) MMD; (**d**) JMMD; (**e**) LMMD; and (**f**) proposed method.

**Figure 8:**
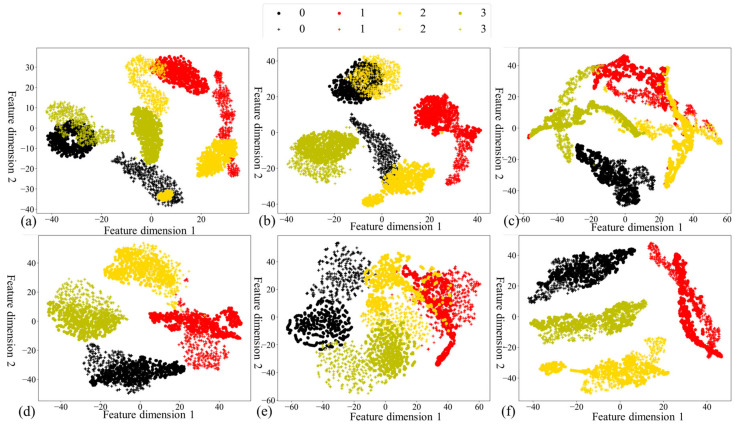
The t-SNE results of: (**a**) DCTLN; (**b**) DASAN; (**c**) MMD; (**d**) JMMD; (**e**) LMMD; and (**f**) proposed method.

In the pursuit of visually elucidating the merits embedded in our proposed methodology, this section employs two crucial tools: confusion matrices and the t-SNE visualization algorithm. Centered on the B→C task, the nuanced insights provided by Figure 7 and Figure 8 unravel the intricacies of the confusion matrices and t-SNE outcomes for the six methods, respectively. The confusion matrix results bring to the fore the capability of our proposed method to accurately predict and identify diverse health conditions. A deeper dive into the t-SNE results unveils the adeptness of our proposed method in harmoniously clustering data originating from both source and target domains, distinguished by shared labels. Of paramount significance are the conspicuously narrower intra-class distances and expansively broader inter-class separations, emblematic of the profound efficacy inherent in our approach. This notable achievement can be attributed to the meticulous integration of discriminative information within the marginal distribution, a unique hallmark of the CKB. Consequently, a palpable elevation in diagnostic performance is discerned. In synthesis, our proposed method unequivocally surmounts extant domain adaptation methodologies.

### 4.6. Ablation Experiment

**Figure 9:**
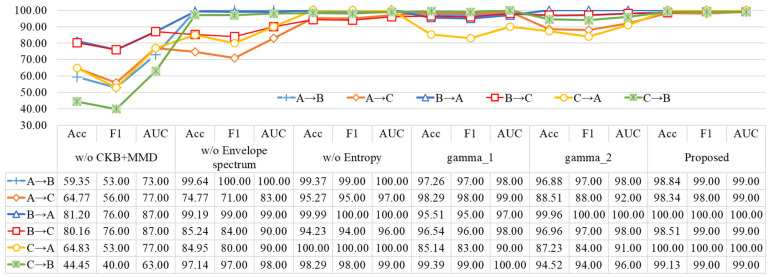
Diagnosis results with different diagnosis models.

A thorough assessment of the rationality and effectiveness of our proposed methodology is conducted through a series of ablation experiments. Following the methodological framework, systematic ablation studies are executed on three critical elements: envelope spectrum analysis, various loss functions, and weight terms. The experimental design encompasses five distinct scenarios: an exclusion of the envelope spectrum, a neglect of the CKB + MMD loss function, an omission of the entropy loss function, a fixation of weight 1 for gamma_2 in Equation (10), and a dynamic adjustment of weight for gamma_1. Iterative repetitions of each experiment (five times) provide a robust basis for result analysis, with the means consolidated and graphically depicted in Figure 9. The results reveal that the model exhibits its least favorable performance when lacking the CKB + MMD loss function. Furthermore, it becomes apparent that entropy, despite its marginal impact, substantiates its essential role in augmenting the diagnostic model’s performance. From this analytical exploration, it is concluded that the structural arrangement of our proposed methodology stands as a rational choice, the selection of pertinent loss functions is judicious, and the proposed method achieves the best diagnostic performance.

### 4.7. Parameter Sensitivity Analysis

**Figure 10:**
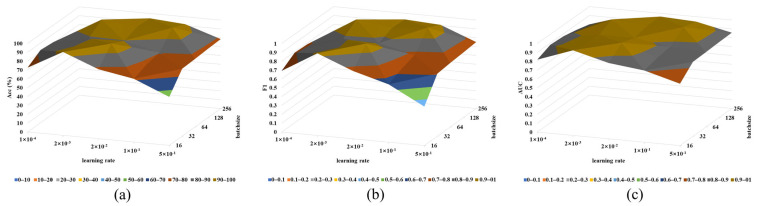
Parameter sensitivity analysis results mapfor (**a**) Case Western Reserve University, (**b**) NASA center’s comprehensive bearing dataset, and (**c**) high-speed traction motor bearing failure data.

To assess the impact of different parameter values on the model, this section conducted parameter sensitivity analysis experiments. Specifically, we analyzed the influence of different learning rates and batch sizes on the performance of the proposed method. The ranges of learning rates and batch sizes were [1 × 10^−4^, 2 × 10^−3^, 2 × 10^−2^, 1 × 10^−1^, 5 × 10^−1^] and [16, 32, 64, 128, 256], respectively, resulting in a total of 25 parameter combinations. The fault diagnosis results under three different performance indicators are shown in Figure 10a,b. It can be observed that different parameter combinations yield different diagnostic results. Based on the experimental results, the optimal parameter combination can be selected. In this study, the learning rate and batch size were chosen as 2 × 10^−3^ and 128, respectively.

## 5. Conclusions

To solve the domain shift in bearing fault diagnosis, this paper proposes a novel cross-device bearing fault diagnosis method based on envelope spectrum analysis and conditional metric learning. Domain shift trials are performed on three different sets of equipment, and the following conclusions can be drawn from the analysis of the experimental results:

(1) Envelope spectrum analysis, as a preprocessing step for the original vibration signals, can highlight fault information and improve the diagnostic accuracy of the model.
(2) The application of the conditional kernel Bures metric aims to boost the efficacy of the model by minimizing the distributional gap. The dynamic weighting mechanism accelerates the model’s optimization process and positively contributes to the improvement of diagnostic accuracy.
(3) In six cross-device transfer tasks, the proposed method outperforms other domain adaptation methods in terms of three performance evaluation metrics, demonstrating stronger diagnostic capability.

## Figures and Tables

**Table 1:** The structure of the proposed network.

Networks Structure	Layer	Parameters Setting
Feature extractor	Conv-1	Kernels 16-64×1, Stride 8, Padding 1; Batch Normalization; ReLU; Maxpooling 2×1, Stride 2
	Conv-2	Kernels 32-3×1, Stride 1, Padding 1; Batch Normalization; ReLU; Maxpooling 2×1, Stride 2
	Conv-3	Kernels 64-3×1, Stride 1, Padding 1; Batch Normalization; ReLU; Maxpooling 2×1, Stride 2
	Conv-4	Kernels 64-3×1, Stride 1, Padding 1; Batch Normalization; ReLU; Maxpooling 2×1, Stride 2
	Conv-5	Kernels 64-3×1, Stride 1, Padding 1; Batch Normalization; ReLU; Maxpooling 2×1, Stride 2
	Conv-6	Kernels 1024-3×1, Stride 1; Batch Normalization; ReLU; Maxpooling 2×1, Stride 2
Classifier	Linear-1	Node: 256
	Linear-2	Node: No.category; Softmax

**Table 2:** The details of the bearing dataset.

Datasets	Bearing Type	Health Condition	Sampling Frequency	Speed, Load	Label
A	Motor bearing	Normal	12 kHz	1750 r/min 2HP	0
		Outer ring fault		1750 r/min 2 HP	1
		Inner ring fault		1750 r/min 2 HP	2
		Roller fault		1750 r/min 2 HP	3
B	Shaft support bearing	Normal	20 kHz	2000 r/min 26.6 kN	0
		Outer ring fault		2000 r/min 26.6 kN	1
		Inner ring fault		2000 r/min 26.6 kN	2
		Roller fault		2000 r/min 26.6 kN	3
C	High-speed traction motors bearing	Normal	10 kHz	2873 r/min 2.87 kN	0
		Outer ring fault		2873 r/min 3.09 kN	1
		Inner ring fault		2766 r/min 2.60 kN	2
		Roller fault		2765 r/min 2.57 kN	3

**Table 3:** The results of different methods.

Datasets	Bearing Type	DCTLN	DASAN	MMD	JMMD	LMMD	Proposed
A→B	Acc	85.69 ± 10.29	77.40 ± 22.73	95.67 ± 3.66	89.06 ± 9.02	86.58 ± 12.86	98.84 ± 1.10
	F1	0.85 ± 0.11	0.74 ± 0.26	0.96 ± 0.04	0.88 ± 0.10	0.85 ± 0.14	0.99 ± 0.01
	AUC	0.90 ± 0.07	0.85 ± 0.15	0.97 ± 0.02	0.93 ± 0.06	0.91 ± 0.09	0.99 ± 0.01
A→C	Acc	62.33 ± 6.22	71.82 ± 17.59	82.90 ± 8.98	71.99 ± 11.90	71.56 ± 16.03	98.34 ± 1.77
	F1	0.53 ± 0.07	0.68 ± 0.23	0.79 ± 0.13	0.66 ± 0.15	0.67 ± 0.19	0.98 ± 0.02
	AUC	0.75 ± 0.04	0.81 ± 0.12	0.89 ± 0.06	0.81 ± 0.08	0.81 ± 0.11	0.99 ± 0.01
B→A	Acc	76.65 ± 18.31	59.58 ± 20.76	96.63 ± 5.12	96.18 ± 4.19	99.09 ± 1.82	100.00 ± 0.00
	F1	0.71 ± 0.25	0.58 ± 0.21	0.97 ± 0.05	0.96 ± 0.05	0.99 ± 0.02	1.00 ± 0.00
	AUC	0.84 ± 0.12	0.73 ± 0.14	0.98 ± 0.03	0.97 ± 0.03	0.99 ± 0.01	1.00 ± 0.00
B→C	Acc	89.73 ± 10.82	75.25 ± 17.17	92.74 ± 10.82	68.76 ± 17.40	81.17 ± 15.03	98.51 ± 1.37
	F1	0.90 ± 0.11	0.69 ± 0.19	0.91 ± 0.15	0.65 ± 0.19	0.78 ± 0.18	0.99 ± 0.01
	AUC	0.93 ± 0.07	0.84 ± 0.11	0.95 ± 0.07	0.79 ± 0.12	0.87 ± 0.10	0.99 ± 0.01
C→A	Acc	93.13 ± 10.91	66.22 ± 11.77	94.98 ± 11.23	84.96 ± 13.73	85.43 ± 13.33	100.00 ± 0.00
	F1	0.91 ± 0.14	0.56 ± 0.15	0.93 ± 0.15	0.80 ± 0.18	0.81 ± 0.17	1.00 ± 0.00
	AUC	0.95 ± 0.07	0.77 ± 0.08	0.97 ± 0.07	0.90 ± 0.09	0.90 ± 0.09	1.00 ± 0.00
C→B	Acc	65.34 ± 14.66	95.33 ± 4.16	83.00 ± 16.23	78.27 ± 14.70	91.52 ± 12.77	99.13 ± 0.60
	F1	0.55 ± 0.20	0.95 ± 0.04	0.80 ± 0.20	0.75 ± 0.17	0.90 ± 0.16	0.99 ± 0.01
	AUC	0.77 ± 0.10	0.97 ± 0.03	0.89 ± 0.11	0.86 ± 0.10	0.94 ± 0.09	0.99 ± 0.00

## Data Availability

Data are contained within the article.
